# Tube Thoracostomy Across Emergency Medicine, Thoracic Surgery, and Intensive Care Residents: A Three-Year Retrospective Analysis of Practice Patterns and Complications at a Tertiary University Hospital

**DOI:** 10.3390/jcm15187292

**Published:** 2026-09-19

**Authors:** Emre Şancı, Rıdvan Atilla, Sefer Özgür, Hamza Çıldır

**Affiliations:** 1 Department of Emergency Medicine, Izmir Bakircay University Cigli Training and Research Hospital, Izmir 35620, Türkiye; 2 Department of Emergency Medicine, European University of Lefka, Mersin 99010, Türkiye; 3 Department of Emergency Medicine, Izmir University of Economics, Izmir 35330, Türkiye; 4 Department of Emergency Medicine, Buca Seyfi Demirsoy Training and Research Hospital, Izmir 35370, Türkiye

**Keywords:** tube thoracostomy, chest tubes, complications, emergency service, hospital, intensive care units, residency

## Abstract

**Background:** Tube thoracostomy is performed by several specialties in several hospital settings, but institution-wide data comparing who performs the procedure, where, how, and with what complications are limited. **Methods:** In this retrospective observational cohort study, all tube thoracostomies performed over a three-year period at a tertiary university hospital were identified through the hospital information system. Procedures were grouped by performing team (emergency medicine, thoracic surgery, and intensive care) and by setting (emergency department [ED], operating room/ward, and intensive care unit [ICU]) and compared with respect to demographics, indications, operator seniority, anesthesia practice, chest tube size, and complications. Effect sizes are reported with 95% confidence intervals (CIs), and predictors of complications were examined in a multivariable binomial logistic regression model. **Results:** In total, 487 procedures were analyzed (76.0% male; median age 56 years). The emergency medicine team performed 47.2% of procedures (all in the ED), the thoracic surgery team 46.8% (the only team active in all three settings), and the intensive care team 6.0%. Indication profiles diverged (*p* < 0.001): traumatic (34.8%) and spontaneous pneumothorax (31.7%) dominated the emergency medicine caseload, pleural effusion (40.4%) the thoracic surgery caseload, and iatrogenic pneumothorax (72.4%) the intensive care caseload. Overall, 95.9% of tubes were 28 F or 32 F; the thoracic surgery team favored 32 F (75.9%; *p* < 0.001). First-year residents performed 39.8% of procedures, two-thirds of them on the thoracic surgery team. Complications occurred in 70 procedures (14.4%), 90.0% of which were tube malpositions. Crude complication rates were 10.0% for emergency medicine, 16.2% for thoracic surgery, and 34.5% for intensive care (*p* = 0.001), but rates rose with the acuity of the setting (ED 10.9%, ICU 26.8%) and within-setting differences between teams were not significant (ED *p* = 0.363; ICU *p* = 0.301). In a multivariable model adjusting for team, setting, age, indication group and operator seniority, neither team (*p* = 0.164) nor setting (*p* = 0.223) was independently associated with complications. Complication rates did not differ by postgraduate year (16.5%, 9.8%, 14.3% and 20.8% for years 0–1 to 3–4; *p* = 0.157, test for trend *p* = 0.738), by tube size (≤28 F 12.0% versus ≥32 F 15.8%; *p* = 0.287) or by age (*p* = 0.276). **Conclusions:** This hospital-wide map of procedural labor shows that the three teams served largely distinct patient populations. Team, setting and indication are so tightly linked in this hospital that no independent effect of specialty could be demonstrated in either direction. Operator seniority and tube size were not associated with complications. Malposition accounted for nine of every ten complications, which supports routine verification of tube position after insertion. Which team drains which pathology, where, and at what level of training is determined locally rather than by specialty training standards, and that map is the principal contribution of this series.

## 1. Introduction

Tube thoracostomy is among the most frequently performed invasive procedures in hospital medicine. A chest tube may be placed emergently to decompress a traumatic pneumothorax or hemothorax, semi-electively to drain a symptomatic pleural effusion or empyema, or urgently at the bedside when mechanical ventilation produces an iatrogenic air leak, and this breadth of indication means that the procedure is performed, in most institutions, by several different specialties working in several different physical settings [1]. In trauma care, timely pleural decompression is a core competency codified in the Advanced Trauma Life Support program [2], while pleural disease guidelines address the elective end of the spectrum, from image guidance to drain selection [3].

Despite its apparent simplicity, tube thoracostomy carries significant morbidity. Reported complication rates range from 1% to 40%, and a meta-analysis of trauma cohorts estimated a pooled rate of 19%, with positional complications forming the largest single category [4]. Individual series illustrate the spread, with overall rates of 30% in a United Kingdom trauma cohort [5], 21% per patient at a level I trauma center [6], and 25% in a prospective single-institution study [7]. Much of this variation reflects heterogeneous definitions and detection methods rather than true differences in practice [4].

Who performs the procedure, and where, has long been suspected to influence these figures, yet the literature is not unanimous. Some investigators found fewer complications when chest tubes were placed by surgeons rather than emergency physicians [6], or identified non-surgical operators as an independent risk factor [7,8], whereas direct comparisons of emergency medicine and surgical residents have found no meaningful difference [9,10], and the setting of insertion has emerged as a determinant in its own right [11]. A limitation of much of this work is its focus on a single setting, usually the emergency department, or a single indication, usually trauma, which leaves the hospital-wide division of labor, namely, which team drains which pathology, where, with what technique, and at what level of training, largely undescribed.

We therefore reviewed all tube thoracostomies performed at our tertiary university hospital over a three-year period. Our aims were to map the distribution of procedures across the three teams that perform them (emergency medicine, thoracic surgery, and intensive care) and the three settings in which they work (emergency department, other (i.e., operating room/ward), and intensive care unit); to characterize the indications, operator seniority, anesthesia practice, and tube size selection of each team; and to compare complication rates across teams and settings.

## 2. Materials and Methods

### 2.1. Study Design and Setting

This retrospective observational cohort study was conducted at a tertiary university hospital. The records of all patients who underwent tube thoracostomy over a three-year period (1 January 2015–1 January 2018) were identified through the Hospital Information Management System and reviewed. Because both the exposure of interest (the team and the setting performing the procedure) and the subsequent outcome (procedure-related complications) were ascertained retrospectively across a defined observation period, the design is a retrospective cohort distinct from a cross-sectional survey. The observation window closed in January 2018. The findings are therefore presented throughout as an analysis of institutional practice patterns rather than as a description of contemporary tube thoracostomy practice.

### 2.2. Procedural Selection, Unit of Analysis, and Data Collection

**Figure 1:**
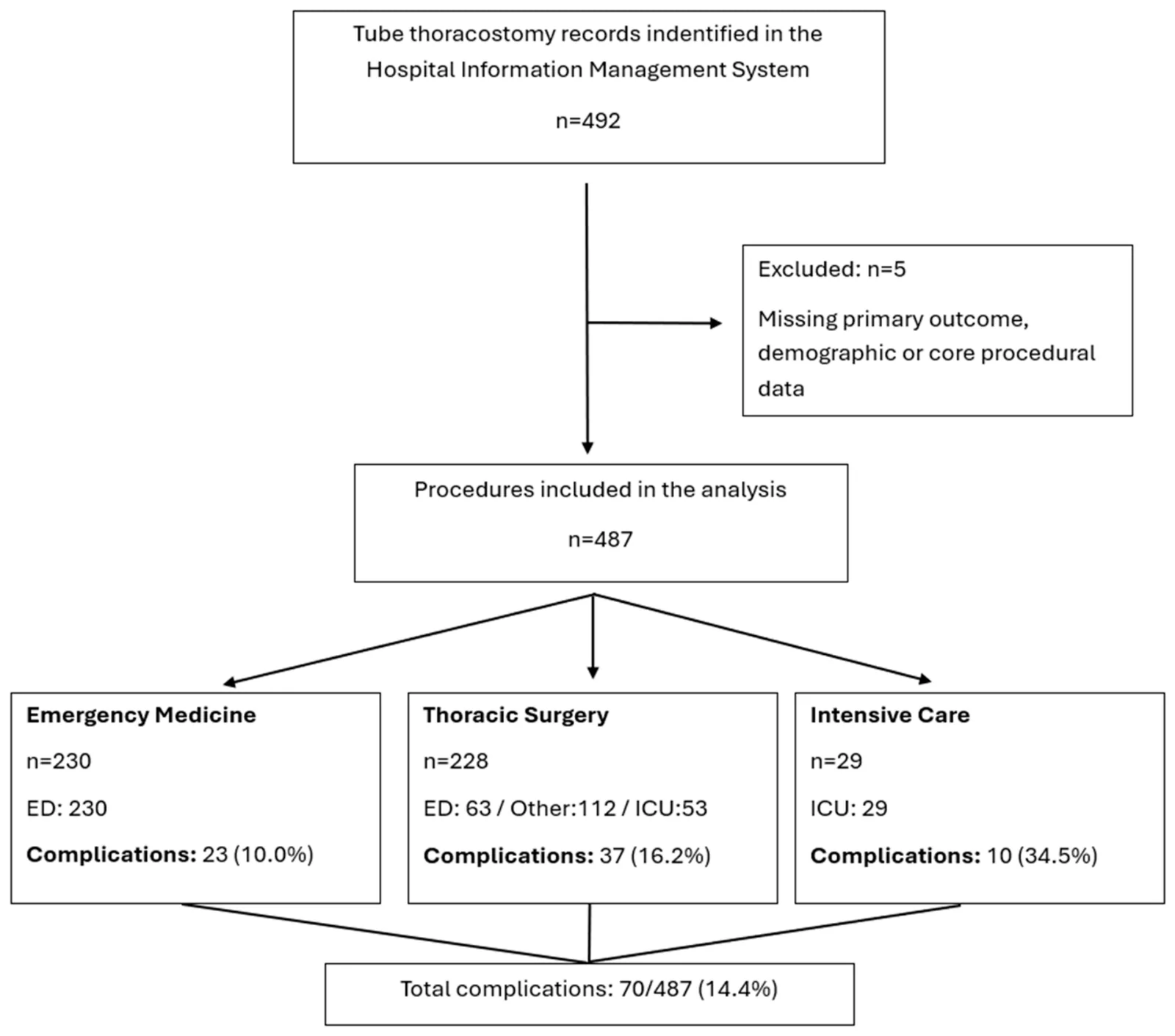
Flow chart of the study.

The Hospital Information Management System query identified 492 tube thoracostomy records for the study period. Five records were excluded because primary outcome data, demographic details, or core procedural characteristics (setting, performing team, indication) could not be retrieved, leaving 487 procedures in the analysis set (Figure 1).

The unit of analysis is the individual procedure, not the individual patient. Terminology has been standardized accordingly: all counts, proportions and comparisons refer to procedures, and the word “patients” is used only for characteristics belonging to the person rather than the procedure, namely age and sex.

For each procedure, demographic and clinical characteristics, the indication for tube thoracostomy, the setting in which it was performed, the specialty of the performing team, the postgraduate year of the resident performing the procedure, the use of local anesthesia, general anesthesia, and sedation, the chest tube size, and procedure-related complications were extracted onto a standard data form. Procedures with no anesthesia or sedation entry were retained in the overall cohort; their handling is described below. Where two indications were documented for the same procedure, the primary indication was used in the analysis. The 487 procedures were contributed by 456 unique patients. 31 patients underwent more than one tube thoracostomy during the study period, with a maximum of 2 procedures per patient. Where a tube was repositioned, exchanged or reinserted because of malposition or another procedure-related problem, the episode was recorded as a single procedure rather than as separate procedures. 29 of the patients had bilateral tube thoracostomy while 2 patients had tube thoracostomy at different times.

Missing data were quantified for every study variable. All core variables including age, sex, performing team, setting, indication, resident postgraduate year, chest tube size and complication status were complete for all 487 included procedures. The only incomplete item was the anesthesia and sedation record: for 28 procedures (5.7%; 23 of them in the emergency department) no local anesthetic, general anesthetic or sedative agent was documented. Furthermore, these procedures were retained in the cohort and are counted within the “Absent” category of tables, so that the denominator of every table remains 487; no imputation was performed and no complete-case exclusion was applied. Because this coding does not distinguish “documented as not administered” from “not documented”, the reported rates of local anesthesia, general anesthesia and sedation should be read as lower bounds.

### 2.3. Definitions

Procedures were classified by setting into three groups: the emergency department (ED), the other departments (i.e., OR/ward), and the intensive care unit (ICU). The residents performing the procedures were likewise classified into three teams: the emergency medicine team, the thoracic surgery team, and the intensive care team. Each procedural team included at least one attending physician together with residents and nurses of the relevant department, and the seniority of the resident performing the procedure was recorded in postgraduate-year bands (0–1, 1–2, 2–3, and 3–4 years). Procedure-related complications comprised tube malposition, infection, minor bleeding and dysrhythmia and were recorded as present or absent for each procedure. The definitions applied are set out below.

Tube malposition was defined radiologically. A tube was classified as malpositioned when the first post-procedural imaging study showed it in a position other than intrapleural and appropriately sited, namely intraparenchymal, intrafissural, or subcutaneous or otherwise extrathoracic; or when the clinical record documented that the tube required repositioning, exchange or removal for positional reasons. The imaging modality was the first post-procedural chest radiograph in every case. No standardized radiological grading scale was applied, and malposition was not graded by severity. Isolated subcutaneous emphysema at the insertion site, in the absence of demonstrable malposition and without any need for repositioning, exchange or removal, was recorded but was not counted as a complication in the primary analysis; a sensitivity analysis counting these procedures is reported in Section 3.7.

Infection was defined as a clinically diagnosed insertion-site or pleural-space infection attributed to the procedure and treated with antibiotics, with drainage, or with both during the hospital stay. Minor bleeding was defined as procedure-related bleeding managed conservatively, without transfusion, surgical exploration or interventional radiological treatment.

Dysrhythmia was defined as a new rhythm disturbance documented during insertion time.

### 2.4. Ethics Approval

The study protocol was approved by the Institutional Ethics Committee of Dokuz Eylül University (decision no. 2018/06-34, date: 1 March 2018). Owing to the retrospective design, the requirement for individual informed consent was waived.

### 2.5. Statistical Analysis

Categorical variables were summarized as counts and percentages, and continuous variables as mean ± standard deviation and median (interquartile range, IQR). The normality of continuous variables was assessed with the Shapiro–Wilk test; as age was not normally distributed, the Mann–Whitney U test was used for two-group comparisons and the Kruskal–Wallis test for three-group comparisons, with Bonferroni-corrected pairwise comparisons. Categorical variables were compared with the Pearson chi-square test; when the expected-count assumption was not met, exact *p* values based on Monte Carlo simulation (100,000 samples) were computed, and Fisher’s exact test was used for 2 × 2 tables. A two-sided *p* value below 0.05 was considered statistically significant.

Because the within-setting comparisons between teams form a family of related tests, the twelve such comparisons (indications, resident seniority, local anesthesia, general anesthesia, sedation and complications, each in the emergency department and in the intensive care unit) were additionally examined after Holm’s step-down correction. Holm-adjusted values are reported wherever they alter the interpretation of the unadjusted result.

For the complication analyses, effect sizes are reported alongside *p* values: odds ratios (OR) with 95% confidence intervals (CI) computed on the log scale by the Woolf method, and absolute risk differences with 95% CI by the normal approximation. The association between operator seniority and complications was assessed both across the four postgraduate-year bands and with a linear-by-linear test for trend.

Predictors of complication were examined in a binomial logistic regression model with complication as the dependent variable. The pre-specified model contained performing team and setting patient age; an indication group contrasting effusive or iatrogenic pleural disease (pleural effusion, empyema, chylothorax, iatrogenic pneumothorax or iatrogenic hemothorax) with traumatic or spontaneous disease; and operator seniority dichotomized as first postgraduate year versus beyond. This gives seven covariate degrees of freedom for 70 events, an events-per-variable ratio of 10.0, at the conventional limit for logistic regression [12]; a more extensively adjusted model, in particular one carrying the full ten-category indication variable, is not supportable at this event count and was not fitted. The contribution of each covariate given the others was assessed by a likelihood-ratio test, collinearity by variance inflation factors, and the structural overlap between team and setting by Cramér’s V. Pre-specified sensitivity analyses were performed: one substituting a four-category indication variable for the binary grouping, and one counting isolated subcutaneous emphysema as a complication. Because only 31 patients contributed more than one procedure (6.4% of procedures), clustering within patients was considered negligible and the procedure was treated as an independent unit of analysis.

Analyses were performed with IBM SPSS Statistics for Windows, version 30.0 (IBM Corp., Armonk, NY, USA); the regression models, effect-size estimates and multiplicity-adjusted *p* values were computed in jamovi version 2.7.38 (The jamovi project, Sydney, Australia).

## 3. Results

Over the three-year study period, 492 tube thoracostomy records were identified and five were excluded for missing core data, leaving 487 procedures for analysis (Figure 1). Of these, 370 (76.0%) were performed on male and 117 (24.0%) on female patients. The median age of the whole cohort was 56 years (interquartile range [IQR] 35–70), the mean age 53.8 ± 21.2 years, and the age range 18–98 years. Female patients were significantly older than male patients (median 64 [IQR 47–75] versus 54 [IQR 31–68] years; *p* < 0.001).

Of the 487 procedures, 230 (47.2%) were performed by the emergency medicine team, 228 (46.8%) by the thoracic surgery team, and 29 (6.0%) by the intensive care team. By setting, 293 procedures (60.2%) were performed in the ED, 112 (23.0%) in the OR/ward (8 in the operating room and 104 on the wards), and 82 (16.8%) in the ICU. The emergency medicine team performed all of its procedures in the ED and the intensive care team performed all of its procedures in the ICU, whereas the thoracic surgery team worked in all three settings (ED: 63, OR/ward: 112, ICU: 53). Of the procedures performed in the ED, 78.5% (230/293) were carried out by the emergency medicine team and 21.5% (63/293) by the thoracic surgery team; of those performed in the ICU, 64.6% (53/82) were carried out by the thoracic surgery team and 35.4% (29/82) by the intensive care team.

The age distribution of the patients differed across teams: the median age was 46 years (IQR 26–65) for the emergency medicine team, 62 years (IQR 48–73) for the thoracic surgery team, and 68 years (IQR 54–81) for the intensive care team (*p* < 0.001). In pairwise comparisons, the patients treated by the emergency medicine team were significantly younger than those of the other two teams (*p* < 0.001 for both), while the thoracic surgery and intensive care teams did not differ from each other (*p* = 0.236). The proportion of female patients also differed across teams (emergency medicine 14.3%, thoracic surgery 34.2%, intensive care 20.7%; *p* < 0.001).

The chest tube sizes used most often were 32 F (300; 61.6%) and 28 F (167; 34.3%); the remaining sizes (22, 24, 26, and 36 F) were chosen in a total of 20 procedures (4.1%). Tube size was not associated with patient sex (*p* = 0.862). Tube size selection did, however, differ significantly across teams (*p* < 0.001): the thoracic surgery team used a 32 F tube in 75.9% of its procedures, compared with 50.4% for the emergency medicine team and 37.9% for the intensive care team (Table 1).

### 3.1. Characteristics of the Indications

Across the whole series, the most frequent indications for tube thoracostomy were traumatic pneumothorax (108; 22.2%), pleural effusion (97; 19.9%), and spontaneous pneumothorax (86; 17.7%), followed by iatrogenic pneumothorax (71; 14.6%) and hemopneumothorax (60; 12.3%). The distribution of indications differed significantly across teams (*p* < 0.001) (Table 2). The emergency medicine team most often intervened for traumatic pneumothorax (80; 34.8%) and spontaneous pneumothorax (73; 31.7%), and the thoracic surgery team for pleural effusion (92; 40.4%) and iatrogenic pneumothorax (44; 19.3%), while iatrogenic pneumothorax accounted for the large majority of the intensive care team’s procedures (21; 72.4%). Viewed by indication, the emergency medicine team performed 84.9% of all procedures for spontaneous pneumothorax, 74.1% of those for traumatic pneumothorax, and 68.3% of those for hemopneumothorax, whereas the thoracic surgery team performed 94.8% of those for pleural effusion.

Among the 293 procedures performed in the ED, the most frequent indications were traumatic pneumothorax (99; 33.8%), spontaneous pneumothorax (78; 26.6%), and hemopneumothorax (52; 17.7%). The indication profiles of the two teams working in this setting differed significantly (*p* < 0.001): the proportion of spontaneous pneumothorax was markedly higher in the emergency medicine team (31.7% versus 7.9%), whereas pleural effusion was more frequent in the thoracic surgery team (19.0% versus 1.7%).

All 112 procedures performed in the OR/ward were carried out by the thoracic surgery team; the leading indications in this setting were pleural effusion (64; 57.1%) and iatrogenic pneumothorax (22; 19.6%). The indications for the 8 procedures performed in the operating room were traumatic hemothorax (3), hemopneumothorax (2), pleural effusion (2), and traumatic pneumothorax (1).

Nearly half of the 82 procedures performed in the ICU were for iatrogenic pneumothorax (40; 48.8%); of these, 21 (52.5%) were performed by the intensive care team and 19 (47.5%) by the thoracic surgery team. The indication profiles of the two teams working in the ICU also differed significantly (*p* = 0.005): iatrogenic pneumothorax predominated among the intensive care team’s procedures (72.4% versus 35.8%), whereas pleural effusion was more frequent among the thoracic surgery team’s procedures (30.2% versus 3.4%).

### 3.2. Seniority of the Performing Residents

Of all procedures, 194 (39.8%) were performed by residents in postgraduate years 0–1, 163 (33.5%) in years 1–2, 77 (15.8%) in years 2–3, and 53 (10.9%) in years 3–4. The seniority distribution differed significantly across teams (*p* < 0.001) (Table 3). The proportion of procedures performed by residents beyond their first year was 72.2% in the emergency medicine team and 43.0% in the thoracic surgery team, whereas all of the intensive care team’s procedures were performed by residents beyond their first year. No first-year resident performed a procedure on the intensive care team, and 82.8% of that team’s procedures were performed by residents in years 3–4. Of the 194 procedures performed by first-year residents, 67.0% belonged to the thoracic surgery team and 33.0% to the emergency medicine team.

In the ED, the proportion of residents beyond their first year was significantly higher in the emergency medicine team than in the thoracic surgery team (72.2% versus 36.5%; *p* < 0.001). In the OR/ward, 51.8% of the thoracic surgery team’s procedures were performed by first-year residents. In the ICU, 60.4% of the thoracic surgery team’s procedures were performed by first-year residents, whereas the seniority distribution of the intensive care team was shifted markedly toward higher seniority (*p* < 0.001).

### 3.3. Local Anesthesia Practice

Local anesthesia was used in 370 procedures (76.0%) overall. Of the procedures performed under local anesthesia, 243 (65.7%) took place in the ED, 95 (25.7%) in the OR/ward, and 32 (8.6%) in the ICU; by setting, the rate of local anesthesia use was 82.9% in the ED, 84.8% in the OR/ward, and 39.0% in the ICU. Within each team’s own procedures, local anesthesia was used in 82.2% by the emergency medicine team, 76.8% by the thoracic surgery team, and 20.7% by the intensive care team (*p* < 0.001); in pairwise comparisons, the emergency medicine and thoracic surgery teams were similar (*p* = 0.497), while the intensive care team’s rate was significantly lower than that of both other teams (*p* < 0.001 for both comparisons) (Table 4).

In the ED, local anesthesia use was similar in the two teams (emergency medicine 82.2%, thoracic surgery 85.7%; *p* = 0.575). The thoracic surgery team’s use of local anesthesia was 85.7% in the ED and 84.8% in the OR/ward but fell to 49.1% in the ICU. In the ICU, the thoracic surgery team used local anesthesia more often than the intensive care team (49.1% versus 20.7%; *p* = 0.017).

### 3.4. General Anesthesia Practice

Ninety procedures (18.5%) were performed under general anesthesia. The rate of general anesthesia was 9.6% in the ED, 12.5% in the OR/ward, and 58.5% in the ICU; all 8 procedures performed in the operating room were carried out under general anesthesia, whereas on the wards the rate remained at 5.8%. Within each team’s own procedures, the rate of general anesthesia was 9.6% for the emergency medicine team, 19.7% for the thoracic surgery team, and 79.3% for the intensive care team, and all pairwise comparisons were significant (overall *p* < 0.001; emergency medicine versus thoracic surgery *p* = 0.007; all other pairwise comparisons *p* < 0.001). Of the 90 procedures performed under general anesthesia, 45 (50.0%) belonged to the thoracic surgery team, 23 (25.6%) to the intensive care team, and 22 (24.4%) to the emergency medicine team (Table 5).

In the ED, the two teams used general anesthesia at similar rates (9.6% versus 9.5%; *p* = 1.000). In the ICU, the intensive care team used general anesthesia significantly more often than the thoracic surgery team (79.3% versus 47.2%; *p* = 0.005).

### 3.5. Sedation Practice

Sedation was used in 255 procedures (52.4%), and every procedure performed with sedation also involved concurrent local anesthesia. The rate of sedation was 79.5% in the ED, compared with 6.2% in the OR/ward and 18.3% in the ICU. Within each team’s own procedures, sedation was used in 79.1% by the emergency medicine team, 30.3% by the thoracic surgery team, and 13.8% by the intensive care team (*p* < 0.001); the emergency medicine team’s use of sedation was significantly higher than that of the other two teams (*p* < 0.001 for both), with no difference between the thoracic surgery and intensive care teams (*p* = 0.240). Of the 255 procedures with sedation, 182 (71.4%) belonged to the emergency medicine team. Within-setting comparisons showed no significant differences between the teams in the ED (79.1% versus 81.0%; *p* = 0.861) or in the ICU (20.8% versus 13.8%; *p* = 0.557) (Table 6).

When anesthesia and sedation practices were considered together, the most frequent approach was local anesthesia combined with sedation (255; 52.4%), followed by local anesthesia alone (114; 23.4%) and general anesthesia alone (89; 18.3%); in one procedure (0.2%), local and general anesthesia were both recorded. No anesthesia or sedation record could be retrieved for 28 procedures (5.7%; 23 of them in the ED).

### 3.6. Complications

Complications occurred in 70 procedures (14.4%) overall. Of these 70 complications, 37 (52.9%) occurred in procedures performed by the thoracic surgery team, 23 (32.9%) in procedures performed by the emergency medicine team, and 10 (14.3%) in procedures performed by the intensive care team.

Of the 293 procedures performed in the ED, 32 (10.9%) were complicated; 23 of these 32 complications (71.9%) occurred in emergency medicine team procedures and 9 (28.1%) in thoracic surgery team procedures. Of the 112 procedures performed in the OR/ward, 16 (14.3%) were complicated, all of them in thoracic surgery team procedures. Of the 82 procedures performed in the ICU, 22 (26.8%) were complicated; 12 (54.5%) occurred in thoracic surgery team procedures and 10 (45.5%) in intensive care team procedures.

Considered by the team, the emergency medicine team had the lowest complication rate, with 23 complications in 230 procedures (10.0%), followed by the thoracic surgery team with 37 in 228 (16.2%), while the intensive care team had the highest rate, with 10 complications in 29 procedures (34.5%) (overall *p* = 0.001) (Table 7). Within individual settings, the differences between the teams did not reach statistical significance (ED: 10.0% versus 14.3%, *p* = 0.363; ICU: 22.6% versus 34.5%, *p* = 0.301); the confidence intervals around these comparisons are wide and are given in Section 3.7 and Table 8.

The most frequent complication was tube malposition, observed in 63 of the 70 complicated procedures (90.0%); the remaining seven comprised infection (4; 5.7%), minor bleeding (2; 2.9%), and dysrhythmia (1; 1.4%).

### 3.7. Factors Associated with Complications

With the emergency medicine team as reference, crude odds of complication were 1.74 times higher for the thoracic surgery team (95% CI 1.00–3.04) and 4.74 times higher for the intensive care team (95% CI 1.97–11.40). The corresponding risk differences were +6.2 percentage points (95% CI +0.1 to +12.4) and +24.5 percentage points (95% CI +6.8 to +42.2).

Thoracic surgery versus emergency medicine in the emergency department gave an odds ratio of 1.50 (95% CI 0.66–3.43). Intensive care versus thoracic surgery in the intensive care unit gave 1.80 (95% CI 0.66–4.89). Both intervals extend from a substantial reduction in odds to a three- to fivefold increase, and neither supports a claim of equivalence between the teams [13].

Between settings, complications were more frequent in the intensive care unit than in the emergency department (OR 2.99, 95% CI 1.62–5.51). The operating room and wards did not differ materially from the emergency department (OR 1.36, 95% CI 0.71–2.59).

Holm’s step-down correction was applied across the twelve within-setting between-team comparisons. In addition, one result changed classification: local anesthesia use by the thoracic surgery and intensive care teams in the intensive care unit (Section 3.3) was no longer significant after correction (unadjusted *p* = 0.017, adjusted *p* = 0.121). All other comparisons retained their original classification. Table 8 reports effect estimates with 95% confidence intervals for all complication comparisons.

When examined individually, no patient or operator characteristic was associated with complications (Table 9). Rates by postgraduate year were 16.5% (32/194) in years 0–1, 9.8% (16/163) in years 1–2, 14.3% (11/77) in years 2–3 and 20.8% (11/53) in years 3–4 (*p* = 0.157), with no linear trend (*p* = 0.738). First-year operators had a complication rate of 16.5% against 13.0% for all others (OR 1.33, 95% CI 0.80–2.21; *p* = 0.293). Rates did not differ by tube size (≤28 F 12.0% versus ≥32 F 15.8%; OR 1.39, 95% CI 0.81–2.38; *p* = 0.287). Patients with a complication were not significantly older than those without (median 60 years, IQR 42–71, versus 55 years, IQR 35–70; *p* = 0.276). Rates by indication were 7.0% for spontaneous pneumothorax, 16.7% for traumatic pleural disease, 15.4% for effusive pleural disease and 15.0% for iatrogenic pleural disease (*p* = 0.186).

The team and setting were almost entirely determined by one another. The emergency medicine team operated only in the emergency department, and the intensive care team only in the intensive care unit. Thoracic surgery was the only team present in more than one setting, and five of the nine possible team-by-setting combinations occurred (Cramér’s V = 0.648; *p* < 0.001). Case mix varied with both. Effusive or iatrogenic pleural disease accounted for 8.3% of the emergency medicine caseload, 67.5% of the thoracic surgery caseload and 82.8% of the intensive care caseload (*p* < 0.001). By setting, it accounted for 13.7% of procedures in the emergency department, 81.3% in the operating room and wards and 80.5% in the intensive care unit. Variance inflation factors in the multivariable model remained below 2.8.

In the multivariable model (Table 8), neither team (*p* = 0.164) nor setting (*p* = 0.223) was independently associated with complications, although each was associated with complications when entered as the sole predictor (team *p* = 0.003; setting *p* = 0.003). Age (*p* = 0.729) and first-year operator status (*p* = 0.191) showed no association. The indication group was the only covariate to reach significance: effusive or iatrogenic disease carried lower adjusted odds of complication than traumatic or spontaneous disease (OR 0.46, 95% CI 0.23–0.95; *p* = 0.036). The unadjusted odds ratio for the same contrast was 1.12 (95% CI 0.67–1.87), so the association emerged only after adjustment.

Both pre-specified sensitivity analyses left the team result unchanged. Replacing the binary indication variable with the four-category version kept the team non-significant (*p* = 0.073) and made indication significant (*p* = 0.006). Counting isolated subcutaneous emphysema as a complication, which raised the event count from 70 to 102, also kept the team non-significant (*p* = 0.168); the setting reached significance in this model (*p* = 0.023). As a collinearity diagnostic, a further model omitting setting showed an association between team and complications (*p* < 0.001; emergency medicine versus thoracic surgery OR 0.48, 95% CI 0.24–0.95; intensive care versus thoracic surgery OR 3.71, 95% CI 1.46–9.42). However, because the team and setting are near-collinear in this cohort, these single-term models cannot attribute the association to either variable.

## 4. Discussion

In this three-year, institution-wide series of 487 tube thoracostomies, two teams, emergency medicine and thoracic surgery, accounted for 94% of all procedures, yet the patients they treated had remarkably little in common. The emergency medicine team drained air, blood, or both from the pleural spaces of younger patients, almost always for spontaneous or trauma-related disease and always in the emergency department. The thoracic surgery team functioned as a hospital-wide consultant service whose workload was dominated by pleural effusion and iatrogenic pneumothorax in patients a median of sixteen years older, and it was the only team active in all three settings. The intensive care team occupied a narrow niche, dealing almost exclusively with iatrogenic pneumothorax in its own units and deploying only its most senior residents to do so. One procedure in seven was complicated; malposition accounted for nine of every ten complications, and although crude complication rates differed significantly between teams, these differences did not persist once setting, case mix, age and operator seniority were accounted for, and the teams, the settings in which they worked and the pathology they drained varied together so closely that no independent contribution of specialty could be identified.

This division of labor is worth documenting in its own right, because it is anything but universal. In our institution the emergency medicine team performed more than three-quarters of the chest tubes inserted in the emergency department, in keeping with the central place of pleural decompression in trauma resuscitation as taught by the Advanced Trauma Life Support program [2]. In another Turkish university hospital, by contrast, thoracic surgery residents performed 79% of emergency department tube thoracostomies [10]. Which specialty carries out the procedure evidently depends less on national training standards than on local staffing, referral culture, and history, a point with practical consequences, since curricula, supervision rules, and quality monitoring must be built around the teams that actually do the work rather than the teams assumed to do it.

Our overall complication rate of 14.4% sits toward the favorable end of the published spectrum, in which rates from 1% to 40% have been reported and a meta-analysis of trauma cohorts estimated a pooled rate of 19% [4]. Individual series illustrate the spread: 30% in a United Kingdom trauma cohort [5], 21% per patient at a level I trauma center [6], 25% in a prospective single-center study [7], 37% in one academic emergency department [14], 24% among nontraumatic insertions in another [15], and as little as 3–4% in a previous Turkish emergency department series [10]. Much of this variation reflects how hard one looks and what one counts rather than how well one operates [4]. The predominance of malposition in our series, at 90% of all complications, is consistent with pooled data in which positional problems form the largest complication subtype [4], and with computed tomography studies that detected malposition in 26% to 42% of tubes after emergency or intensive care insertion [16,17,18], most of it invisible on plain radiographs [8,16,19]. Because our ascertainment relied on routine documentation and radiography rather than systematic cross-sectional imaging, the true malposition rate in this cohort was almost certainly higher than the figure we report.

At first glance the crude rates invite a ranking of the teams: 10.0% for emergency medicine, 16.2% for thoracic surgery, and 34.5% for intensive care (*p* = 0.001). We would caution against reading this as a hierarchy of procedural skill, because the teams were not performing the same procedure on the same patients. The thoracic surgery team’s patients were substantially older, and its indication profile was dominated by pleural effusion, empyema, and iatrogenic disease; in other words, by chronic or complicated pleural pathology in which adhesions, loculation, and prior intervention distort the pleural space and predispose to exactly the complication that dominated our series, tube malposition. Complicated or localized pleural disease has been identified as an independent predictor of tube thoracostomy complications in the emergency department, in an analysis in which operator specialty and seniority carried no independent weight [15], and in trauma cohorts, physiological compromise and the severity of the thoracic injury show the same pattern [6,20]. Our own multivariable model reproduces that null result for operator specialty, but it diverges from Rossetto and colleagues on the direction of the pathology effect: they found localized pleural disease to raise the odds of complication, whereas in our cohort, effusive or iatrogenic disease carried lower adjusted odds. The emergency medicine team, by contrast, worked almost exclusively on younger patients with spontaneous or traumatic pneumothorax, that is, on a pleural space that is usually structurally normal. Case mix is an intuitively attractive explanation for the gap between the teams’ figures, and it was the explanation we initially favored, but our own data do not bear it out in the form we expected. Furthermore, when indication was entered into a multivariable model alongside team, setting, age and seniority, effusive or iatrogenic pleural disease carried lower adjusted odds of complication than traumatic or spontaneous disease (OR 0.46, 95% CI 0.23–0.95), and the crude rate for spontaneous pneumothorax, the emergency medicine team’s second most frequent indication, was the lowest of any group at 7.0%, while traumatic disease carried the highest at 16.7%. No objective measure of procedural difficulty was available to us—prior ipsilateral surgery, imaging-confirmed loculation, adhesions, body habitus and coagulation status were all absent from the dataset—so we cannot exclude that a better measure of pleural complexity would behave as the adhesion-and-loculation account predicts. What we can say is that the indication categories we do have do not support it. Two further observations bear on the comparison. First, in the emergency department, the only setting in which the two teams worked side by side, their complication rates did not differ significantly (10.0% versus 14.3%; *p* = 0.363); the confidence interval (OR 1.50, 95% CI 0.66–3.43) is nevertheless wide enough to accommodate both a halving and a threefold increase in risk, so this does not show the teams to be equivalent. Crucially, nor were the two caseloads clinically comparable even within that setting: spontaneous pneumothorax accounted for 31.7% of the emergency medicine team’s procedures there against 7.9% of the thoracic surgery team’s, and pleural effusion for 1.7% against 19.0% (*p* < 0.001). Sharing a physical setting did not make the patients alike. Second, the thoracic surgery team’s own rate was 14.3% in the emergency department and 14.3% in the operating room and wards, rising to 22.6% in the intensive care unit, although the operators belonged to the same team throughout.

The crude gradient across settings ran alongside the gradient across teams instead of replacing it: complications rose from 10.9% in the emergency department to 14.3% in the operating room and wards and 26.8% in the intensive care unit, while the corresponding team rates rose from 10.0% to 16.2% and 34.5%. Because the emergency medicine team worked only in the emergency department and the intensive care team only in the intensive care unit, these two gradients are largely the same gradient observed twice, and neither can be given precedence over the other in these data. Notably, the intensive care team recorded the highest crude rate of all (34.5%, based on only 29 procedures) despite fielding the most senior operators in the study, with 82.8% of its procedures performed by residents in their final training year. Seniority, in other words, did not neutralize the environment, an observation that echoes earlier work in which resident seniority did not predict chest tube complications [8,15]. The intensive care unit concentrates the circumstances under which tube thoracostomy is least forgiving: positive-pressure ventilation, emergent iatrogenic air leaks, edematous tissue, restricted patient positioning, and pleural spaces already altered by disease or prior intervention. Etoch and colleagues observed similar associations three decades ago, linking complications to shock, intensive care admission, and mechanical ventilation as opposed to injury scores [6], and a prospective computed tomography study found malposition in 30% of chest tubes inserted in the critically ill [17]. The literature on setting is not unidirectional; one urban series found inpatient ward insertions faring worse than emergency department insertions [11], while another identified emergency department placement itself as an independent predictor of complications [21], which argues for local audit rather than extrapolation. In our institution the setting in which the tube was inserted, the pathology it was inserted for and the specialty of the operator moved together, and when all three were modeled simultaneously, none emerged as an independent predictor. We are therefore unable to say which of them carries the risk, and these data do not support a claim that setting matters more than specialty.

The seniority findings carry a further message for training. Two of every five procedures in the series were performed by residents in their first postgraduate year, and two-thirds of those first-year procedures belonged to the thoracic surgery team, whose ward workload in particular functioned as an early-training proving ground: more than half of the OR/ward insertions were performed by first-year residents. There is nothing inherently wrong with this, since tube thoracostomy is precisely the kind of index procedure a surgical residency should teach early, but the combination of the most junior operators with the most anatomically distorted pleural spaces deserves institutional attention. Deneuville attributed much of the morbidity of the procedure to inadequate training of those who perform it [7], and Ball and colleagues, having documented resident complications occult to plain radiography, argued for structured instruction and closer supervision [8]. Contemporary pleural guidance points in the same direction, emphasizing competence-based training, thoracic ultrasound where applicable, and verification of tube position after insertion [22]. Complication rates did not differ across postgraduate-year bands (16.5%, 9.8%, 14.3% and 20.8% from year 0–1 to year 3–4; *p* = 0.157), showed no linear trend with seniority (*p* = 0.738), and did not separate first-year operators from the rest (16.5% versus 13.0%; OR 1.33, 95% CI 0.80–2.21); first-year status contributed nothing to the multivariable model (*p* = 0.191). This is consistent with earlier work in which resident seniority did not predict chest tube complications [8,15], and it means that the concentration of junior operators on the wards, although a legitimate object of training policy, is not shown by these data to be a source of harm. Simulation-based teaching, explicit supervision thresholds for first-year residents outside the resuscitation area, and a low threshold for cross-sectional imaging when a tube underperforms remain defensible on general grounds, but we present them as quality-improvement considerations prompted by the distribution of procedures rather than as findings established by this study.

Tube size selection was conservative by contemporary standards. Ninety-six percent of all tubes were 28 F or 32 F; small-bore devices were essentially absent, and the thoracic surgery team showed the strongest large-bore preference, choosing 32 F in three-quarters of its procedures. The evidence accumulated over the past decade points the other way. In trauma, 28–32 F tubes performed no worse than 36–40 F tubes [23]; a randomized trial found 14 F pigtail catheters as effective as 28 F tubes for uncomplicated traumatic pneumothorax, with less insertion-site pain [24]; meta-analyses support small-bore catheters as initial treatment for pneumothorax [25]; and pooled data suggest comparable failure rates even in traumatic hemothorax [26]. Current British Thoracic Society guidance now favors smaller-bore drains for most nontraumatic indications [27], with its companion clinical statement addressing the practicalities of insertion [22]. Our data describe a practice largely untouched by this shift, and they cannot test it. Within the narrow range of calibers actually used, we found no association between tube size and complications (≤28 F 12.0% versus ≥32 F 15.8%; *p* = 0.287), but with 95.9% of tubes at 28 F or 32 F and genuine small-bore devices essentially absent, this comparison says nothing about pigtail or other small-bore catheters, which were not used in this cohort. The case for smaller-bore drainage in these indications therefore rests entirely on the external evidence cited above and not on any result generated within this series. The same qualification applies to our suggestion of a low threshold for cross-sectional imaging, which follows from the dominance of malposition in the complication profile and from the published computed tomography literature [16,17,18,19], not from any comparison of imaging strategies made here.

Anesthesia and analgesia practice tracked the setting rather than the team. In the emergency department, the two teams behaved almost identically, using local anesthesia in more than four-fifths of procedures and sedation in about four-fifths, with no significant between-team differences, which suggests that within a shared environment, practice converges regardless of specialty. General anesthesia was essentially confined to the operating room and the intensive care unit, where most patients were presumably already intubated and sedated, and this also explains the low rates of separately documented local anesthesia there. Two details merit attention. Sedation was never used without concurrent local anesthesia, which is reassuring, since pleural guidelines emphasize adequate local anesthesia as the foundation of procedural analgesia [3,22]. Less reassuring is that no anesthesia or sedation record could be retrieved for 5.7% of procedures; for a procedure consistently experienced as one of the most painful performed at the bedside [24], documentation of analgesia is not a bureaucratic nicety but part of its quality.

A final consideration concerns the age of the dataset. These procedures were performed between January 2015 and January 2018, and while standard pleural practice has not moved much since, bedside thoracic ultrasound, image-guided insertion and small-bore pigtail catheters have all become more prominent, and current British Thoracic Society guidance favors smaller-bore drainage for most non-traumatic indications [22,27]. Our dataset contains no record of ultrasound guidance, because that variable was not collected, and almost no small-bore catheters. We therefore make no claim that the procedural patterns reported here represent contemporary practice, at our institution or elsewhere.

What the series documents, and what we believe retains value independently of drain technology, is the hospital-wide division of procedural labor: which team drains which pathology, in which setting, and at what level of training. That division is set by staffing structure, referral culture and the organization of training instead of by catheter design, and it is the aspect of our findings least likely to have been overturned by the shift toward smaller-bore devices. We accordingly present this work as a historical institutional practice-pattern analysis, and its complication figures as a baseline against which subsequent local audit can be measured.

### Limitations

This study has the limitations inherent to its design. It is retrospective and single-center, and both the division of labor between the teams and the complication rates we report are shaped by local staffing and referral patterns; they describe our institution and may not transfer elsewhere. We also acknowledge that the direction of several findings—that complicated pleural pathology in older, often ventilated patients carries greater procedural risk than spontaneous pneumothorax in young patients—is already well established [6,15,20]. The contribution we claim is descriptive: a hospital-wide map of who performs this procedure, where, for what, and at what stage of training, which is rarely reported in full and cannot be inferred from single-setting or single-indication series. Patients with incomplete records were excluded, introducing a potential selection bias. The dataset contained no injury severity scores, physiological scores, comorbidity indices, body habitus, coagulation status, or ventilation status, so residual confounding by indication cannot be excluded, and we have deliberately avoided causal interpretation of the between-team differences. Complications were recorded as present or absent, without standardized severity grading or timing, which limits comparability with series using structured classifications [4]. Finally, the intensive care team contributed only 29 procedures, so its complication estimate is imprecise, and anesthesia records were missing for 5.7% of procedures.

Three limitations deserve to be stated more strongly than the rest, because they bear directly on the study’s central comparison.

First, the complication outcome is, to a first approximation, a measure of radiographically detected tube position: malposition accounted for 63 of the 70 complications (90.0%). Ascertainment depended on routine documentation and routine chest radiography instead of protocolized cross-sectional imaging, so malposition was certainly under-detected relative to what systematic computed tomography would have found [16,17,18,19] and, more seriously, was probably under-detected to different degrees in different settings, because critically ill patients undergo imaging more often and more thoroughly. We were unable to retrieve the number and type of imaging studies performed per procedure and therefore cannot quantify this differential or offer the sensitivity analysis it would require. The likely direction of the bias is nonetheless clear: it would inflate the apparent complication rate in the intensive care unit, which is precisely where our highest rates were observed. This is the principal threat to the validity of the setting comparison, and we regard it as a limitation of the outcome measure itself rather than as a general caveat.

Second, performing team, setting and indication are so closely tied to one another in this hospital that their effects can be separated only weakly. As reported in Section 3.7, only five of the nine possible team-by-setting combinations occurred, and the proportion of effusive or iatrogenic pleural disease ranged from 8.3% to 82.8% across teams. Although variance inflation factors in our model remained below 2.8, the model is estimated largely from the thoracic surgery team’s presence in all three settings, and with 70 events and seven covariate degrees of freedom, it sits at the conventional events-per-variable limit [26]. Its non-significant findings for team and setting should therefore be read as an inability to demonstrate an effect, not as a demonstration of its absence [13]; no statement in this paper attributes the observed differences to specialty, to setting, or to case mix rather than to the others.

Third, postgraduate-year bands were used to compare seniority across three specialties whose curricula, procedural exposure and competency milestones differ. A first-year thoracic surgery resident and a first-year emergency medicine resident are not necessarily equivalent in chest-tube experience, and the seniority comparisons in Table 3 should be read as a description of who performed the procedures instead of as a calibrated comparison of operator experience. Our null findings for seniority must be interpreted in that light, and it is further limited by the absence of any record of the level of supervision under which each procedure was carried out.

## 5. Conclusions

Tube thoracostomy at our institution is a genuinely multi-specialty procedure, but the specialties do not share a caseload: emergency medicine drains the young, the emergent, and the traumatic; thoracic surgery drains the older and pleurally complex, in every setting of the hospital; and intensive care drains its own ventilated patients. One procedure in seven was complicated, and malposition accounted for nearly all of it. Crude complication rates differed between the teams, but neither team nor setting remained associated with complications after adjustment for one another, for age, for indication and for operator seniority, and neither seniority nor tube size was associated with complications at all. Because team, setting and pathology vary almost completely together in this hospital, these data cannot establish an independent effect of specialty, in either direction, and the crude differences should be read as a map of where procedural risk is concentrated in this cohort rather than as a ranking of operators. The practical yield of that map is narrow but real: malposition accounted for nine of every ten complications, which supports routine verification of tube position after insertion, with a low threshold for cross-sectional imaging when a tube underperforms. Recommendations about supervision of junior residents and about small-bore drainage are quality-improvement considerations drawn from the wider literature, not findings of this study; these are historical data from 2015 to 2018 and describe neither contemporary practice nor any tested comparison between small- and large-bore drainage. Prospective, severity-adjusted, multicenter data, with protocolized post-procedural imaging and a design in which operator specialty is not determined by setting, would be needed to put the between-team question to a proper test.

## Figures and Tables

**Table 1:** Distribution of chest tube sizes by sex and by performing team.

	22 F	24 F	26 F	28 F	32 F	36 F	Total	*p*
Gender								
Female	1 (0.9)	3 (2.6)	0	40 (34.2)	73 (62.4)	0	117	0.862
Male	1 (0.3)	10 (2.7)	2 (0.5)	127 (34.3)	227 (61.4)	3 (0.8)	370	
Performing team								
Emergency medicine	1 (0.4)	9 (3.9)	2 (0.9)	101 (43.9)	116 (50.4)	1 (0.4)	230	<0.001
Thoracic surgery	0	4 (1.8)	0	50 (21.9)	173 (75.9)	1 (0.4)	228	
Intensive care	1 (3.4)	0	0	16 (55.2)	11 (37.9)	1 (3.4)	29	
Total	2 (0.4)	13 (2.7)	2 (0.4)	167 (34.3)	300 (61.6)	3 (0.6)	487	

Values are numbers and (row %). F: French.

**Table 2:** Distribution of tube thoracostomy indications by setting and by performing team.

Setting	Team	Spontaneous PTX	Traumatic PTX	Traumatic HTX	Hemopneumothorax	Pleural Effusion	Empyema	Chylothorax	Iatrogenic PTX	Iatrogenic HTX	Tension PTX	Total	*p*
ED	Emergency medicine	73 (31.7)	80 (34.8)	15 (6.5)	41 (17.8)	4 (1.7)	8 (3.5)	0	6 (2.6)	1 (0.4)	2 (0.9)	230	<0.001
	Thoracic surgery	5 (7.9)	19 (30.2)	6 (9.5)	11 (17.5)	12 (19.0)	4 (6.3)	1 (1.6)	3 (4.8)	1 (1.6)	1 (1.6)	63	
	ED total	78 (26.6)	99 (33.8)	21 (7.2)	52 (17.7)	16 (5.5)	12 (4.1)	1 (0.3)	9 (3.1)	2 (0.7)	3 (1.0)	293	
Other (OR, ward)	Thoracic surgery	2 (1.8)	7 (6.3)	6 (5.4)	5 (4.5)	64 (57.1)	3 (2.7)	0	22 (19.6)	2 (1.8)	1 (0.9)	112	
ICU	Thoracic surgery	3 (5.7)	2 (3.8)	5 (9.4)	1 (1.9)	16 (30.2)	3 (5.7)	1 (1.9)	19 (35.8)	3 (5.7)	0	53	0.005
	Intensive care	3 (10.3)	0	0	2 (6.9)	1 (3.4)	0	0	21 (72.4)	2 (6.9)	0	29	
	ICU total	6 (7.3)	2 (2.4)	5 (6.1)	3 (3.7)	17 (20.7)	3 (3.7)	1 (1.2)	40 (48.8)	5 (6.1)	0	82	
Overall	Emergency medicine	73 (31.7)	80 (34.8)	15 (6.5)	41 (17.8)	4 (1.7)	8 (3.5)	0	6 (2.6)	1 (0.4)	2 (0.9)	230	<0.001
	Thoracic surgery	10 (4.4)	28 (12.3)	17 (7.5)	17 (7.5)	92 (40.4)	10 (4.4)	2 (0.9)	44 (19.3)	6 (2.6)	2 (0.9)	228	
	Intensive care	3 (10.3)	0	0	2 (6.9)	1 (3.4)	0	0	21 (72.4)	2 (6.9)	0	29	
	Total	86 (17.7)	108 (22.2)	32 (6.6)	60 (12.3)	97 (19.9)	18 (3.7)	2 (0.4)	71 (14.6)	9 (1.8)	4 (0.8)	487	

Values are numbers and (row %). ED: emergency department; OR: operating room; ICU: intensive care unit; PTX: pneumothorax; HTX: hemothorax.

**Table 3:** Distribution of the performing resident’s postgraduate seniority by setting and by team.

Setting	Team	0–1 Years	1–2 Years	2–3 Years	3–4 Years	Total	*p*
ED	Emergency medicine	64 (27.8)	91 (39.6)	52 (22.6)	23 (10.0)	230	<0.001
	Thoracic surgery	40 (63.5)	17 (27.0)	5 (7.9)	1 (1.6)	63	
	ED total	104 (35.5)	108 (36.9)	57 (19.5)	24 (8.2)	293	
Other (OR, ward)	Thoracic surgery	58 (51.8)	42 (37.5)	8 (7.1)	4 (3.6)	112	—
ICU	Thoracic surgery	32 (60.4)	10 (18.9)	10 (18.9)	1 (1.9)	53	<0.001
	Intensive care	0	3 (10.3)	2 (6.9)	24 (82.8)	29	
	ICU total	32 (39.0)	13 (15.9)	12 (14.6)	25 (30.5)	82	
Overall	Emergency medicine	64 (27.8)	91 (39.6)	52 (22.6)	23 (10.0)	230	<0.001
	Thoracic surgery	130 (57.0)	69 (30.3)	23 (10.1)	6 (2.6)	228	
	Intensive care	0	3 (10.3)	2 (6.9)	24 (82.8)	29	
	Total	194 (39.8)	163 (33.5)	77 (15.8)	53 (10.9)	487	

Values are numbers and (row %). ED: emergency department; OR: operating room; ICU: intensive care unit.

**Table 4:** Use of local anesthesia by setting and by performing team.

Setting	Team	Present	Absent	Total	*p*
ED	Emergency medicine	189 (82.2)	41 (17.8)	230	0.575
	Thoracic surgery	54 (85.7)	9 (14.3)	63	
	ED total	243 (82.9)	50 (17.1)	293	
Other (OR, ward)	Thoracic surgery	95 (84.8)	17 (15.2)	112	—
ICU	Thoracic surgery	26 (49.1)	27 (50.9)	53	0.017
	Intensive care	6 (20.7)	23 (79.3)	29	
	ICU total	32 (39.0)	50 (61.0)	82	
Overall	Emergency medicine	189 (82.2)	41 (17.8)	230	<0.001
	Thoracic surgery	175 (76.8)	53 (23.2)	228	
	Intensive care	6 (20.7)	23 (79.3)	29	
	Total	370 (76.0)	117 (24.0)	487	

Values are numbers and (row %). ED: emergency department; OR: operating room; ICU: intensive care unit.

**Table 5:** Use of general anesthesia by setting and by performing team.

Setting	Team	Present	Absent	Total	*p*
ED	Emergency medicine	22 (9.6)	208 (90.4)	230	1.000
	Thoracic surgery	6 (9.5)	57 (90.5)	63	
	ED total	28 (9.6)	265 (90.4)	293	
Other (OR, ward)	Thoracic surgery	14 (12.5)	98 (87.5)	112	—
ICU	Thoracic surgery	25 (47.2)	28 (52.8)	53	0.005
	Intensive care	23 (79.3)	6 (20.7)	29	
	ICU total	48 (58.5)	34 (41.5)	82	
Overall	Emergency medicine	22 (9.6)	208 (90.4)	230	<0.001
	Thoracic surgery	45 (19.7)	183 (80.3)	228	
	Intensive care	23 (79.3)	6 (20.7)	29	
	Total	90 (18.5)	397 (81.5)	487	

Values are numbers and (row %). ED: emergency department; OR: operating room; ICU: intensive care unit.

**Table 6:** Use of sedation by setting and by performing team.

Setting	Team	Present	Absent	Total	*p*
ED	Emergency medicine	182 (79.1)	48 (20.9)	230	0.861
	Thoracic surgery	51 (81.0)	12 (19.0)	63	
	ED total	233 (79.5)	60 (20.5)	293	
Other (OR, ward)	Thoracic surgery	7 (6.2)	105 (93.8)	112	—
ICU	Thoracic surgery	11 (20.8)	42 (79.2)	53	0.557
	Intensive care	4 (13.8)	25 (86.2)	29	
	ICU total	15 (18.3)	67 (81.7)	82	
Overall	Emergency medicine	182 (79.1)	48 (20.9)	230	<0.001
	Thoracic surgery	69 (30.3)	159 (69.7)	228	
	Intensive care	4 (13.8)	25 (86.2)	29	
	Total	255 (52.4)	232 (47.6)	487	

Values are numbers and (row %). ED: emergency department; OR: operating room; ICU: intensive care unit.

**Table 7:** Frequency of complications by setting and by performing team.

Setting	Team	Present	Absent	Total	*p*
ED	Emergency medicine	23 (10.0)	207 (90.0)	230	0.363
	Thoracic surgery	9 (14.3)	54 (85.7)	63	
	ED total	32 (10.9)	261 (89.1)	293	
Other (OR, ward)	Thoracic surgery	16 (14.3)	96 (85.7)	112	—
ICU	Thoracic surgery	12 (22.6)	41 (77.4)	53	0.301
	Intensive care	10 (34.5)	19 (65.5)	29	
	ICU total	22 (26.8)	60 (73.2)	82	
Overall	Emergency medicine	23 (10.0)	207 (90.0)	230	0.001
	Thoracic surgery	37 (16.2)	191 (83.8)	228	
	Intensive care	10 (34.5)	19 (65.5)	29	
	Total	70 (14.4)	417 (85.6)	487	

Values are numbers and (row %). ED: emergency department; OR: operating room; ICU: intensive care unit.

**Table 8:** Effect sizes with 95% confidence intervals for the complication comparisons, and adjusted odds ratios from the multivariable logistic regression model.

Comparison	Complication Rate	Odds Ratio (95% CI)	Risk Difference, (95% CI)	*p*
Between teams, whole cohort				
Thoracic surgery vs. emergency medicine	16.2% vs. 10.0%	1.74 (1.00–3.04)	+6.2 (+0.1 to +12.4)	0.053
Intensive care vs. emergency medicine	34.5% vs. 10.0%	4.74 (1.97–11.40)	+24.5 (+6.8 to +42.2)	0.001
Intensive care vs. thoracic surgery	34.5% vs. 16.2%	2.72 (1.17–6.31)	+18.3 (+0.3 to +36.2)	0.023
Between teams, within the same setting				
ED: thoracic surgery vs. emergency medicine	14.3% vs. 10.0%	1.50 (0.66–3.43)	+4.3 (−5.2 to +13.8)	0.363
ICU: intensive care vs. thoracic surgery	34.5% vs. 22.6%	1.80 (0.66–4.89)	+11.8 (−8.8 to +32.5)	0.301
Between settings				
Other (OR, ward) vs. ED	14.3% vs. 10.9%	1.36 (0.71–2.59)	+3.4 (−4.0 to +10.8)	0.390
ICU vs. ED	26.8% vs. 10.9%	2.99 (1.62–5.51)	+15.9 (+5.7 to +26.1)	<0.001
ICU vs. Other (OR, ward)	26.8% vs. 14.3%	2.20 (1.07–4.52)	+12.5 (+1.0 to +24.1)	0.043
Multivariable model (complication ~ team + setting + age + indication group + seniority)				
Emergency medicine vs. thoracic surgery	-	0.65 (0.28–1.55)	-	0.335
Intensive care vs. thoracic surgery	-	2.40 (0.81–7.09)	-	0.113
Other (OR, ward) vs. ED	-	1.49 (0.57–3.91)	-	0.414
ICU vs. ED	-	2.47 (0.87–6.99)	-	0.089
Age, per 10 years	-	1.02 (0.89–1.17)	-	0.729
Effusive or iatrogenic pleural disease	-	0.46 (0.23–0.95)	-	0.036
Operator in first postgraduate year	-	1.47 (0.83–2.61)	-	0.191

CI: confidence interval; ED: emergency department; OR: operating room; ICU: intensive care unit.

**Table 9:** Complication rates by operator seniority, chest tube size, indication group, and age.

Factor	Procedures, *n*	Complications, *n* (%)	*p*
Operator seniority (postgraduate year)			
0–1 years	194	32 (16.5)	0.157
1–2 years	163	16 (9.8)	
2–3 years	77	11 (14.3)	
3–4 years	53	11 (20.8)	
First year vs. beyond first year	194 vs. 293	16.5% vs. 13.0%	0.293
Chest tube size			
≤28 F	184	22 (12.0)	0.287
≥32 F	303	48 (15.8)	
Indication group			
Spontaneous pneumothorax	86	6 (7.0)	0.186
Traumatic pleural disease	204	34 (16.7)	
Effusive pleural disease	117	18 (15.4)	
Iatrogenic pleural disease	80	12 (15.0)	
Age			
Complicated vs. uncomplicated, median (IQR)	—	60 (42–71) vs. 55 (35–70)	0.276

IQR: interquartile range; F: French.

## Data Availability

The dataset analyzed during the current study is available from the corresponding author on reasonable request.
